# New Therapeutic Horizons for Graves’ Hyperthyroidism

**DOI:** 10.1210/endrev/bnaa022

**Published:** 2020-08-26

**Authors:** Laura C Lane, Tim D Cheetham, Petros Perros, Simon H S Pearce

**Affiliations:** 1 Translational and Clinical Research Institute, Newcastle University, Newcastle-upon-Tyne, UK; 2 Endocrine unit, Royal Victoria Infirmary, Newcastle-upon-Tyne Hospitals NHS Foundation Trust, Newcastle-upon-Tyne, UK; 3 Department of Paediatric Endocrinology, The Great North Children’s Hospital, Newcastle-upon-Tyne, UK

**Keywords:** Graves’ disease, hyperthyroidism, thyroid hormone, thyroid-stimulating hormone receptor, immunomodulation, immunotherapy

## Abstract

Graves’ hyperthyroidism is characterized by the presence of autoantibodies that stimulate the thyroid-stimulating hormone receptor (TSHR), resulting in uncontrolled secretion of excessive thyroid hormone. Conventional treatments, including antithyroid medication, radioiodine, or surgery have remained largely unchanged for the past 70 years and either lack efficacy for many patients, or result in lifelong thyroid hormone replacement therapy, in the case of the latter 2 options. The demand for new therapeutic options, combined with greater insight into basic immunobiology, has led to the emergence of novel approaches to treat Graves’ hyperthyroidism. The current therapies under investigation include biologics, small molecules, and peptide immunomodulation. There is a growing focus on TSHR-specific treatment modalities, which carry the advantage of eliciting a specific, targeted approach, with the aim of avoiding disruption of the functioning immune system. These therapies present a new opportunity to supersede the inadequate treatments currently available for some Graves’ patients, offering hope of successful restoration of euthyroidism without the need for ongoing therapy. Several of these therapeutic options have the potential to translate into clinical practice in the near future. This review provides a comprehensive summary of the recent advances and various stages of development of the novel therapeutic approaches to treat Graves’ hyperthyroidism.

## Graphical Abstract

**Graphical Abstract:**
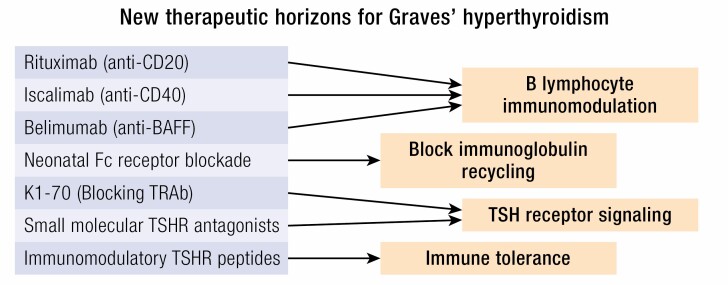

**ESSENTIAL POINTS**

- The conventional therapeutic options for Graves’ disease have not improved over the past 70 years, despite substantial unmet clinical need and a significant lack of efficacy for many patients.
- The demand for innovative therapeutic options has led to the emergence of novel approaches in the treatment of Graves’ hyperthyroidism, including biologic, small molecule, and peptide immunomodulation, several of which have translational potential into clinical practice in the near future.
- Targeting the direct cause of hyperthyroidism in Graves’ disease using TSHR-specific modalities, has a clear advantage in providing a targeted approach while avoiding disruption of a functioning immune system.
- The introduction of novel therapeutics may lead to the restoration of a euthyroid state without the requirement for ongoing therapy, but the potential risk of immunocompromise and cost implications needs careful consideration.

The therapeutic options available for patients with Graves’ hyperthyroidism have remained largely unchanged for the past 70 years, despite the current treatments having either limited efficacy or significant drawbacks. The classical approach to treating Graves’ hyperthyroidism involves the administration of antithyroid drugs to block thyroid hormone synthesis, or alternatively, destruction or removal of the thyroid by radioiodine or surgery (1). Unfortunately, relapse occurs in over half of adults after stopping antithyroid drugs, and the lack of functional thyroid tissue following definitive treatment with radioiodine or surgery results in hypothyroidism with a lifelong requirement for levothyroxine replacement and associated clinical and biochemical monitoring. Patients with active Graves’ orbitopathy (GO) are frequently managed with high-dose glucocorticoids, a treatment that has also been the mainstay of therapy for more than 50 years (2). Unfortunately, significant patient dissatisfaction with outcome and a reduced quality of life remains a concern despite improved surgical rehabilitation (3), and advances in this field are the subject of a recent review (4). This review summarizes the emergence of novel targeted therapies that are paving the way for a new era in the treatment of Graves’ hyperthyroidism.

## Pathophysiology

Graves’ hyperthyroidism is an autoimmune condition that arises as a result of the loss of immunological tolerance to the thyroid-stimulating hormone receptor (TSHR) (1). Elevated circulating thyroid hormones in Graves’ hyperthyroidism arise because of stimulating TSHR autoantibodies (TRAbs), which bind to leucine-rich repeats in the extracellular domain of the TSHR located on the surface of the thyrocytes (5). This mimics the action of thyroid-stimulating hormone (TSH; thyrotropin) resulting in excessive, autonomous thyroid hormone production and hyperplasia of thyroid epithelial cells. The mature TSHR is a G protein–coupled receptor found primarily on thyroid follicular cells. It comprises an entirely extracellular A-subunit, and a B-subunit consisting of a small extracellular leader, 7 transmembrane domains, and an intracellular tail (5). The carboxy-terminal part of the A-subunit participates in a flexible hinge region; TSH binding to the leucine-rich repeats of the A-subunit results in a conformational change transduced through this hinge region, leading to a pivot or toggle of the 7 transmembrane domains. Part of this hinge region may be viewed as an auto-ligand or internal agonist which is displaced in response to both TSH and TRAb binding. Following the structural change in the transmembrane domains, Gsα signaling to increase 3′,5′-cyclic adenosine monophosphate (cAMP) and Gq signaling to activate phospholipase pathways, results in thyroid hormone synthesis and secretion (5). Interestingly, certain TSHR mutations may selectively abrogate Gq signaling without affecting the GSa-cAMP signal (6). It is known that TSHRs exist as homomultimeric complexes in certain situations (7), but whether this is influenced by ligand binding, or is necessary for signaling is under active investigation.

Histologically, the thyroid gland in Graves’ hyperthyroidism demonstrates diffuse follicular cell hyperplasia with varying extents of lymphocytic infiltration into the thyroid stroma (8). These infiltrates consist of both T- and B lymphocytes and may form ectopic intrathyroidal germinal centers, creating an important source of the pathogenic TRAb-producing plasma cells (9). Extrathyroidal TRAb production has been found at regional bone marrow and lymph nodes sites (10), explaining why TRAbs, albeit at reduced concentrations, persist following surgical removal of the thyroid gland (11).

The TSHR is also found in orbital fibroblasts and the upper dermis where binding of TRAbs results in a proliferative response that contributes to the extrathyroidal signs seen in Graves’ hyperthyroidism, GO, and pretibial myxedema (5). Clinical manifestations of GO affect about 25% of people diagnosed with Graves’ hyperthyroidism, and it can be facially disfiguring, may cause functional visual disabilities, and carries the potential for occasional loss of sight (12).

Although the specific immune mechanisms driving Graves’ hyperthyroidism remain unknown, a greater understanding of the interactions between B and T lymphocytes and the autoantigen-antibody complex has enabled progressive insights into the immunopathology underpinning Graves’ hyperthyroidism (5). The role of B cells in Graves’ hyperthyroidism is well established, not only in their capacity as immunoglobulin-secreting plasma cells producing the pathogenic TRAb autoantibody, but also in their ability to act as antigen-presenting cells, likely presenting TSHR epitopes to T cells to perpetuate disease, as well as modulating the immune response by producing both pro- and anti-inflammatory cytokines and chemokines (13).

**Figure 1.**
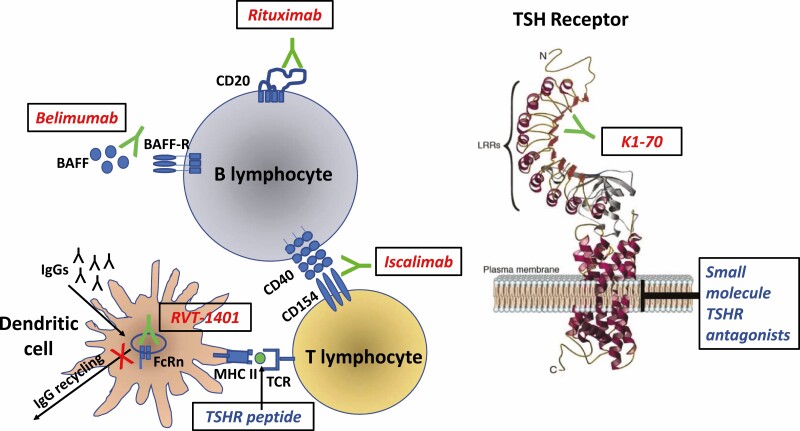
Illustration of novel therapeutic approaches in the treatment of Graves’ hyperthyroidism. The therapeutics in red indicate those that are monoclonal antibodies. Abbreviations: BAFF, B-cell activating factor; BAFF-R, B cell activating factor receptor; FcRn, neonatal immunoglobulin Fc receptor; IgGs, immunoglobulins; K1-70, TSHR-blocking antibody; MHC II, major histocompatibility complex class II; RVT-1401, FcRn blocker; TCR, T cell receptor; TSHR, thyroid-stimulating hormone receptor. TSH receptor image reprinted by permission from Springer Nature: Immunologic Research. Morshed SA, Latif R, Davies TF. Delineating the autoimmune mechanisms in Graves’ disease. 2012.

The described immunological insights have led to the emergence of several novel therapeutic options, most of which remain under investigation in clinical or preclinical studies (Table 1). In addition, Graves’ hyperthyroidism, in contrast to many other autoimmune conditions, has a specific autoantigen which is the extracellular domain of the TSHR. Thus, the TSHR provides an ideal therapeutic candidate for targeted immunomodulation. Current therapeutic options for the treatment of Graves’ hyperthyroidism that are discussed below include those directly targeting the B cells or their associated interactors and cytokines, or alternatively, specific TSHR modulation by the use of small molecule antagonists, antagonistic TSHR monoclonal antibodies, or “tolerogenic” TSHR peptides (Fig. 1). The advantages and disadvantages of the current and novel therapeutic options for Graves’ hyperthyroidism are described in Table 2.

**Table 1.** Summary of the Novel Therapeutic Approaches Being Investigated and Their Potential or Proven Efficacy in the Treatment of Graves’ Hyperthyroidism

Mechanism	Novel therapies	Stage of development	Potential efficacy in GH	Proven efficacy in GH
B-cell depletion	Rituximab (*Anti-CD20 mAb)*	Phase 2 trials (14, 15)	+	+ (*)
Blocking CD40 receptor interactions *(blocks CD40–CD154 co-stimulatory pathway, attenuating B cell activation)*	Iscalimab (CFZ533) (*Anti-CD40 mAb)*	Phase 2 trial (16)	+++	++ (*)
Blocking FcRn-IgG interactions *(inhibits IgG recycling)*	RVT-1401 Rozanolixizumab Efgartigimod	Phase 2 trial (RVT-1401, NCT03922321)	+++	ND
Blocking BAFF interaction (*reduces B cell proliferation and survival)*	Belimumab (*Anti-BAFF mAb)*	Phase 2 trial (EudraCT 2015-002127-26)	++	ND
Small molecule TSHR antagonists *(directly inhibit TSHR signaling)*	ANTAG-3 VA-K-14 S37a	Preclinical (17-20)	+++	ND
TSHR-blocking antibodies (*blocks TSHR stimulation by TSH or TRAbs*)	K1-70 (*Anti-TSHR mAb*)	Case report (21) Phase 1 trial (NCT02904330)	+++	++
TSHR-specific immunotherapy (*induces tolerogenic immune response*)	ATX-GD-59 (*TSHR peptide*)	Phase I trial (22)	+++	++ (*)

+, ++, +++; strength of potential or proven efficacy based on current available evidence

Abbreviations: BAFF, B-cell activating factor; FcRn, neonatal immunoglobulin Fc receptor; GH, Graves’ hyperthyroidism; mAb, monoclonal antibody; ND, no data; TSHR, thyroid-stimulating hormone receptor; TRAbs; TSH receptor autoantibodies.

(*) in those with low pretreatment TRAb levels

**Table 2.** Advantages and Disadvantages of Therapeutic Options in Graves’ Hyperthyroidism

Therapy	Advantages	Disadvantages
Antithyroid drugs (CBZ, PTU, MMI)	Noninvasive, oral tablet	High risk of recurrence
	Outpatient therapy	Frequent monitoring/clinic attendance
	No radiation hazard or surgical risk	Risk of minor (eg, rash, urticaria, arthralgia) or major side effects (eg, agranulocytosis, hepatotoxicity, vasculitis)
Radioiodine	Definitive treatment	Radiation exposure
	Outpatient procedure	Risk of exacerbation/development of GO
	Few adverse effects	Permanent hypothyroidism
	Cost effective	Need to delay pregnancy and avoid breastfeeding
	Reduces goiter size	Radiation safety precautions
Thyroidectomy	Definitive treatment	Inpatient procedure
	Effective	Requires anesthetic
		Permanent hypothyroidism
		Surgical complications (RLN damage, HPT)
		Scarring
		Postoperative pain
B-cell therapies	*Avoids side effects associated with ATD	**Cost implications
	*Potential for restoration of euthyroidism without ongoing therapy	**Uncertain effect on long-term hypothyroidism, goiter size, and prevention of relapse
	May benefit GO	Infusion side effects
		Risk of infection
		Most effective in those with low TRAb levels
		Potential thromboembolic events (iscalimab)
		Increased psychiatric events (belimumab)
TSHR-targeted therapies	As above* and:	As above**
	No global immunosuppression	
	May benefit GO	
	*Small molecule TSHR antagonists:*	
	May be active in oral form	
	May be effective independent of TRAb concentration	
TSHR peptide desensitization	As above* and:	As above**
	No global immunosuppression	Potential disease exacerbation in susceptible individuals
	May benefit GO	Given subcutaneously (bruising/swelling at injection site)

Abbreviations: ATD, antithyroid drug; CBZ, carbimazole; GO, Graves’ orbitopathy; HPT, hypoparathyroidism; MMI, methimazole; PTU, propylthiouracil; RLN, recurrent laryngeal nerve; TRAb, thyroid-stimulating hormone receptor autoantibodies.

## Novel Therapeutic Strategies

### B-lymphocyte depletion (CD20 depletion)

As a humorally driven condition, the essential role of B cells in Graves’ hyperthyroidism provides a logical therapeutic target for immunomodulatory treatment. The B-cell depleting therapy rituximab (RTX) has been used for more than 20 years to treat lymphoproliferative malignancies, such as lymphoma, with increasing use over the past decade to treat autoimmune disease. Although the anti-CD20 monoclonal antibody RTX is the most widely studied of the B-cell therapies, the precise mechanisms by which RTX produces a beneficial effect remain uncertain. Direct apoptosis, complement-mediated cytotoxicity, and antibody-dependent cellular cytotoxicity all appear to contribute to RTX efficacy in depleting B cells (23). The CD20 transmembrane protein is expressed on B-lineage lymphocytes, from early pre-B to mature and memory B cells; however, CD20 expression is lost during B-cell differentiation into mature antibody-secreting plasma cells. Circulating B-lymphocyte depletion is very rapid, occurring within a few hours of RTX infusion. The survival of long-lived plasma cells (CD20-negative) and refractory memory B cells in lymphoid tissues explains how RTX-treated individuals are still able to mount an immune response against naturally encountered and previously vaccinated pathogens (24). Although the reasons remain unclear, memory B cells appear to be more resistant to depletion with RTX compared to naïve B cells, allowing them to be recruited in a secondary immune response (25).

RTX was originally reported to have efficacy in controlling rheumatoid arthritis, with subsequent studies in myasthenia gravis (MG), anti-neutrophil cytoplasmic antibody (ANCA)-associated vasculitis, systemic lupus erythematosus (SLE) and multiple sclerosis (26-30). Several groups have investigated the use of RTX in Graves’ hyperthyroidism. Fassi et al undertook a prospective study of 20 patients with Graves’ hyperthyroidism comparing short-term treatment using methimazole with or without RTX. Although they demonstrated some efficacy with sustained remission in the RTX group (4/10 patients) after a mean of 23 months follow-up, it appeared most effective in those with low TRAb levels at presentation (<5 IU/L) (14). Another small prospective study by Heemstra et al, including 13 patients with recurrent Graves’ hyperthyroidism, demonstrated that 70% remained euthyroid after RTX treatment with significantly decreased TRAb and free thyroxine levels, after a mean follow-up duration of 18 months (15). Similarly, the patients who responded had low pretreatment TRAb levels (median 4 IU/L) and less severe hyperthyroidism.

Some, but not all, studies have shown a reduction in serum TRAb antibody levels following RTX therapy (14, 15, 31), but the reduction does not correlate with the extent of B-cell depletion achieved (15, 32), nor does the effect seem to be greater than that expected with antithyroid medication alone (14). Additionally, in contrast to the previous prospective studies specifically investigating the effect of RTX on hyperthyroidism, several studies, including 2 RCTs, have examined the effect of RTX on GO (33, 34). Although studies show a mixed outcome (4, 33, 34), the clinical improvement of GO seen with RTX appears to occur independently of thyroid function or circulating thyroid antibodies (15, 31, 32, 35-37).

Most patients will have complete (>99%) B-cell depletion following a single 500mg dose of RTX, and this persists for 5 to 20 months before recovery of the peripheral B-cell compartment (38), with recent evidence suggesting that much lower doses of RTX (100 mg) are equally effective (39). The vast majority of residual peripheral B cells exhibit a plasma cell or memory cell phenotype (38); however, complete intrathyroidal B cell depletion has been demonstrated following RTX therapy (40). The persistence of residual autoreactive memory B cells alongside plasma cells, which may continue to produce the pathogenic TRAb autoantibody, may contribute to the lack of definitive success in treating Graves’ hyperthyroidism with RTX. A detailed assessment of the immunological response in patients with type 1 diabetes suggests that RTX does not reset early B-cell tolerance checkpoints and that this may be why durable remission does not occur (41). The results of a clinical trial investigating RTX therapy in 27 young (12-20 years of age) patients with Graves’ hyperthyroidism are awaited (ISRCTN20381716; the RIGD study); however, current evidence does not support the routine use of RTX in Graves’ hyperthyroidism in adults.

Adverse effects have been reported with RTX, the most frequent of which is a mild infusion reaction including throat itching and nasal congestion, which resolves on slowing the infusion with or without the administration of hydrocortisone. There have also been reports of articular and gastrointestinal symptoms, specifically colitis, related to circulating immune complexes following RTX (42). The rare complication of progressive multi-focal leukoencephalopathy has largely been reported in patients with predisposing comorbidities who have received multiple immunosuppressive agents (43). An increased risk of serious infection has been reported in RTX-treated patients, but this tends to occur in patients with concomitant severe immunodeficiency or those with underlying malignancy (44). RTX has been used for many years in the treatment of various autoimmune diseases, and a review over 9.5 years involving repeated courses of RTX in rheumatoid arthritis patients demonstrated no evidence of an increased safety risk or increased reporting rates of any type of adverse events compared with placebo plus methotrexate (45). In addition, the vast majority of studies using RTX in Graves’ hyperthyroidism or GO have demonstrated no serious adverse events (15, 31-33, 36, 37). Newer second-generation CD20-depleting strategies, including ocrelizumab and ofatumumab, which theoretically have lower immunogenicity and improved tolerability (46, 47), may provide a future option for Graves’ patients.

### Disruption of B-cell activation or activity

#### Blocking CD40 interactions

CD40, a tumor necrosis factor (TNF) family receptor found on thyrocytes and antigen-presenting cells, including B cells, has a primary role in coordinating effective antigen presentation (48). Its ligand CD154 (CD40 ligand; CD40L) is transiently expressed on activated T cells and other nonimmune cells under inflammatory conditions. The CD40–CD154 interaction initiates a co-stimulatory pathway that provides the second signal required for the initiation of an adaptive humoral immune response (1). This interaction between B and T lymphocytes is proposed to have a central role in the pathogenesis of Graves’ hyperthyroidism as it is required for intrathyroidal germinal center formation, and for maturing the B-cell repertoire to allow production of pathogenic thyroid-stimulating antibodies (1, 9, 49).

Variants in the *CD40* gene have been associated with several autoimmune diseases, including Graves’ hyperthyroidism, where they appear to influence thyroid antibody production and provide a predictive marker of relapse (50-53). Functional studies have demonstrated that the disease-associated variant in CD40 alters the consensus Kozak initiation sequence, resulting in increased translational efficiency and suggesting that overexpression of CD40 has a causative association with the predisposition to Graves’ hyperthyroidism (54). Indeed, several different murine models have confirmed that either genetic or chemical modulation of CD40 signaling can modify the severity of autoimmune thyroiditis or thyroid autoantibody production (49, 55, 56), establishing CD40 as a potential therapeutic target in the treatment of Graves’ hyperthyroidism.

The anti-CD40 monoclonal antibody iscalimab (CFZ533) targets the CD40–CD154 co-stimulatory pathway, resulting in attenuation of the B-cell activation signal (57). Iscalimab is a nondepleting (Fc silent) antibody, designed to block the CD40 receptor interactions without the removal of CD40-expressing cells. Of the autoimmune conditions, it has been best studied in Sjögren’s syndrome, where an RCT demonstrated that iscalimab was well-tolerated and led to clinical improvement (58).

In a recent open-label study, 15 adult patients with untreated Graves’ hyperthyroidism were given 5 doses of intravenous iscalimab over a 12-week period (16). During the 24-week follow-up period, 7 (47%) patients were deemed “responders” with normal free triiodothyronine and free thyroxine levels and without a need for additional antithyroid medication during this period. In addition, TRAb concentrations were significantly reduced following iscalimab treatment, with 4 (27%) patients achieving normal TRAb levels by week 20, all of whom were “responders.” Those that did not respond were noted to have higher baseline TRAb levels, to have a larger goiter, and to be more likely to use cigarettes and have GO. CD40 engagement by iscalimab decreased total cell-surface CD40 protein on peripheral B cells by 40%, persisting for at least 8 weeks after the last dose was administered. Levels of serum CXCL13, a chemokine with an essential role in germinal center activity, also significantly declined following treatment (16). The decreased CXCL13 concentrations may inhibit the formation of ectopic intrathyroidal germinal centers in Graves’ hyperthyroidism. Indeed, CD40 blockade has been demonstrated to inhibit ectopic lymphoid structures in inflamed tissue in the murine model of Sjögren’s syndrome (59).

Iscalimab was found to be safe and well-tolerated, with no serious treatment-related adverse events reported. Similar to RTX, iscalimab is an immunosuppressive therapy and therefore risk of infection is always a concern. CD40 is expressed on vascular endothelium and platelets, so thromboembolic complications have been highlighted as another hypothetical risk—although this was not observed in the Graves’ disease patients treated. Unfortunately, after the 24-week follow-up period, 4 of the 7 “responders” relapsed and 3 of these patients required low-dose antithyroid medication.

Targeted blockade of the CD40–CD154 interaction shows promising results in Graves’ hyperthyroidism, at least in the short-term and in those with lower TRAb levels (<20 IU/L). The fact that CD40 receptors are also present on orbital fibroblasts suggests the potential for a beneficial effect on GO as well. Indeed, 2 patients with GO whose hyperthyroidism responded to iscalimab also had improvement of eye symptoms and signs (16). An RCT of longer duration to assess the possibility of sustained remission is now warranted.

#### Blocking immunoglobulin recycling (FcRn therapeutics)

The long half-life associated with IgG antibodies, such as TRAbs, is attributed to the neonatal immunoglobulin Fc receptor (FcRn), which recycles endocytosed IgG antibody by binding it in the acidic conditions of the lysosome and recycling it to the cell membrane for release back into the circulation (60). FcRn-deficient mice have shown resistance to autoimmune disease (61), and blockade of FcRn has resulted in the amelioration of autoimmune disease in different animal models (62, 63). Therefore, inhibition of FcRn is an attractive novel therapeutic concept for IgG-mediated autoimmune diseases like Graves’ hyperthyroidism, where acclerated antibody catabolism and the associated reduction in the levels of circulating pathogenic TRAb would be benefical.

The 2 most widely studied compounds that target FcRn are efgartigimod and rozanolixizumab (64), both of which are currently in phase 3 studies for autoimmune disease. Efgartigimod is a human IgG_1_-derived Fc fragment, while rozanolixizumab is a humanized, IgG_4_ anti-FcRn monoclonal antibody, both of which block FcRn-IgG interactions, thereby inhibiting IgG recycling and accelerating the removal of pathogenic IgG autoantibodies from the circulation (60, 65). The therapeutic effect of intravenous immunoglobins in several autoimmune diseases is also mediated by functional Fc receptor blockade, but these novel FcRn therapeutics demonstrate increased receptor binding affinity, which results in efficacy at a much lower dose (less than 50 mg/kg vs 1-2 g/kg body weight) (66, 67). Both efgartigimod and rozanolixizumab caused a sustained reduction in circulating IgG levels of approximately 75% to 90%, both in preclinical studies of murine models of autoimmune disease (arthritis and encephalitis) (63, 68) and in healthy human subjects (64, 69).

Efgartigimod is currently being investigated in MG, immune thrombocytopenia (ITP), and pemphigus vulgaris. In a phase 2 RCT of efgartigimod in 12 patients with generalized MG, a rapid decrease of up to 70% in total IgG and anti-acetylcholine receptor autoantibody levels was demonstrated, alongside a rapid and sustained clinical improvement in 75% of those treated, compared with 25% of the placebo group (70). Similar findings were demonstrated in a phase II trial in ITP with a reduction in total IgG level associated with significantly increased platelet count and reduced bleeding (71). Rozanolixizumab has also undergone phase 2 trials in patients with ITP and MG, demonstrating almost a 70% mean reduction of serum IgG and IgG autoantibodies associated with clinical response (72). These studies have demonstrated both efgartigimod and rozanolixizumab to be well-tolerated, with a mild headache being the most frequently described side effect and no treatment-related serious or severe adverse events reported (70-72).

Other potential Fc and FcγR-targeting therapeutics for autoimmune disease are in various phases of development from preclinical to phase 2 studies (64). Although neither rozanolixizumab nor efgartigimod have been investigated in Graves’ hyperthyroidism, a phase 2 trial with another biologic FcRn blocker, RVT-1401, is about to report (NCT03922321) and it seems likely that these approaches are worthy of further exploration in Graves’ hyperthyroidism.

### Inhibition of B-cell proliferation and differentiation (blockade of B-cell activating factor)

B-cell activating factor (BAFF) is a member of the TNF family of cytokines and has an essential role in B-lymphocyte activation, differentiation, and survival. Elevated circulating BAFF levels have been found in patients with several autoimmune conditions, including active Graves’ hyperthyroidism, where both degree of elevation of thyroid hormones and TRAb concentrations have been demonstrated to correlate with serum BAFF levels (73). Genetic variants in BAFF have also been associated with susceptibility to Graves’ hyperthyroidism (74, 75). Therefore, BAFF is a logical therapeutic target molecule for B-cell–driven autoimmune conditions such as Graves’ hyperthyroidism.

The BAFF monoclonal antibody belimumab binds to and antagonizes the biological activity of soluble BAFF. Blocking the interaction of BAFF with its receptor negatively effects B-cell proliferation, indirectly decreasing B-cell survival and reducing production of autoantibodies (76). The increased thyroidal expression of BAFF and its primary receptor, BAFF-R, in the infiltrating immune cells and thyrocytes in patients with Graves’ hyperthyroidism suggests a central role of the BAFF–BAFF-R interactions in its pathogenesis (77). With this rationale, belimumab treatment is currently under investigation in an RCT conducted in Graves’ hyperthyroidism with active orbitopathy (EudraCT 2015-002127-26).

Efficacy of belimumab alone, or in combination with RTX, has been demonstrated in SLE, with significant improvements in long-term organ damage (78, 79). Conversely, belimumab was not found to be efficacious in patients with ANCA-associated vasculitis (80). Belimumab is currently licensed for treatment of seropositive SLE and, although it has been found generally safe and well-tolerated with no increased risk of serious infection (78, 80), an increased risk of psychiatric events has been reported by the UK Medicines and Healthcare products Regulatory Agency in patients with SLE on belimumab.

As discussed above, the isolated use of a B-cell depleting therapy (eg, RTX) to treat Graves’ hyperthyroidism may lack efficacy due to the persistence of long-lived plasma cells and residual memory cells. Furthermore, following B-cell depletion there is a surge in plasma BAFF concentration. Concurrent targeting of both the homeostatic factors that are responsible for B-cell proliferation and differentiation, along with a B-cell depleting therapy may enhance the effect on tissue-resident autoreactive B cells.

### TSHR-specific modalities

As the direct cause of hyperthyroidism in Graves’ disease is stimulation of the TSHR, several groups have been developing approaches that directly prevent TSHR signaling, either through small molecules or by using antibodies that block receptor activation. Furthermore, TSHR peptides are being investigated to see if they may have long-lasting immunomodulatory properties. A key advantage of this strategy when compared to the immunomodulatory strategies described above is their more specific, targeted approach which—from a theoretical standpoint—is unlikely to have a deleterious impact on the patient’s ability to fight infection.

#### Small molecule TSHR antagonists

Small molecule agonists and antagonists have the potential to directly stimulate or inhibit TSHR signaling that could lead to highly potent therapies for thyroid dysfunction. A series of compounds that inhibit TSHR function as inverse agonists (inhibiting basal as well as agonist-induced signaling) have been developed; the best studied compound has been termed ANTAG-3 (17, 18). These compounds inhibit TSH-stimulated cAMP production in vitro and lower thyroid hormone levels in mice treated with the thyroid-stimulating monoclonal antibody M22, suggesting likely efficacy in inhibiting TRAb-induced Graves’ hyperthyroidism. Two other TSHR antagonist compounds, VA-K-14 and S37a, have been identified by high-throughput library screening and both are able to inhibit TSH- and patient TRAb-induced signaling in vitro (19, 20). A key issue is the structural homology between the TSHR, and the follicle stimulating hormone receptor and lutenizing hormone/chorionic gonadotropin receptors, which gives rise to the potential for off-target reproductive effects. VA-K-14 gives minor antagonism of FSH signaling in vitro, whereas S37a appears to be more specific. Elegant structural studies have shown that S37a binds TSHR in the juxta-membrane region between the C-terminal end of the extracellular domain, which has an internal agonist function, and the first extracellular loop (81). VA-K-14 and ANTAG3 most likely fit within a hydrophobic pocket that more directly stabilizes the transmembrane domains in an “off” conformation (17, 19). This modeling suggests that all 3 of these compounds interact with TSHR at sites that are distinct from the extracellular domain site of both TSH and TRAb binding, meaning that they should be effective irrespective of circulating TRAb concentration. These small molecule compounds are expected to be active orally, and because of their precise targeting to the TSHR they are anticipated to be suitable for long-term administration. None of these compounds have been trialed in humans yet, but this approach may hold promise for both long-term control of hyperthyroidism without the need for thyroid ablation and for the amelioration of thyroid eye disease.

#### TSHR-blocking antibodies

Occasional patients with GO may present with active inflammatory eye disease in the presence of hypothyroidism and high titers of TRAb antibodies, which contain a proportion of TSHR-blocking antibodies. Sanders et al were able to clone a monoclonal TSHR-blocking antibody, termed K1-70 from such a patient (82). In rats, K1-70 was able to completely suppress the serum thyroxine rise following administration of the stimulating M22 antibody, suggesting potential efficacy in Graves’ hyperthyroidism with high serum TRAb levels (83). K1-70 has been given on compassionate grounds to a patient whose metastatic follicular thyroid cancer was being driven by TRAb antibodies associated with co-existing GO (21). The response in terms of thyroid-stimulating activity in the serum was dramatic; cancer progression was initially arrested, and eye disease improved markedly and in a sustained fashion. Thus, this treatment clearly holds promise for both Graves’ hyperthyroidism and GO. There is an ongoing phase 1 trial (NCT02904330) of K1-70 in treatment-naïve Graves’ patients, which is due to report in the next year.

#### TSHR-specific immunotherapy

Conventional approaches to combating autoimmune disease involve using drugs with broad immunosuppressive effects and hence the potential side effect of susceptibility to infection. An alternative approach that has been successfully used to treat allergic disease is to desensitize the immune response to the specific allergen (immune stimulant) (84). The principle that administering small and increasing doses of soluble antigen can lead to tolerogenic immune responses has now also been demonstrated in several autoimmune diseases, leading to a new therapeutic approach (85). However, there is a concern that administration of an antigen in a susceptible individual could lead to exacerbation of disease, as seen in some preclinical settings (86). Repeated administration of tolerogenic peptides from the insulin molecule has been successful in reducing HbA1c and insulin requirements in newly diagnosed adults with type 1 diabetes (87). In a similar approach, Jansson et al identified dominant TSHR T-cell epitopes. These were administered to HLA-DR3 transgenic mice that had been primed for hyperthyroidism by TSHR cDNA immunization, and the TSHR peptide-pretreated mice showed a profound reduction in induced TRAbs and lower thyroid hormone levels (88). In a phase 1 study, this TSHR peptide mixture, termed ATX-GD-59, was then administered every 2 weeks subcutaneously to patients with treatment-naïve Graves’ hyperthyroidism, leading to normalization of hyperthyroidism in 5 of 10 patients and improvement in 7 of 10 (22). TRAb levels improved in line with reducing thyroid hormone levels. This novel treatment may have very little in the way of side effects except for swelling and bruising at the injection site. Phase 2 studies are awaited.

### Limitations

The described novel therapeutic approaches are not without potential limitations. As well as cost implications and the potential risk of immunocompromise with the non–TSHR-specific therapies, it remains unclear whether they will ameliorate the long-term risk of hypothyroidism, reduce goiter, or indeed prevent a late relapse of Graves’ hyperthyroidism. It seems likely that patients with Graves’ hyperthyroidism will continue to be at risk for thyroid dysfunction after cessation of therapy, necessitating annual biochemical monitoring.

## Conclusion

Conventional treatment of Graves’ hyperthyroidism with surgery, radioiodine, or antithyroid drugs has not substantially changed since the late 1940s. An abundance of new therapeutic approaches, involving biologic, small molecule, and peptide immunomodulation are currently at different stages of development and several will translate into the clinic over the next few years. These therapies may render destructive radioiodine thyroid ablation and thyroidectomy obsolete treatments of historical interest, although the advantages of restoring a euthyroid state without the need for ongoing therapy will need to be balanced against potential risks such as immunocompromise. Future studies need to focus on understanding the most effective combinations of the conventional medical and surgical therapies with these recently introduced options.

## Data Availability

Data sharing is not applicable to this article as no datasets were generated or analyzed during the current study.

## Abbreviations

ANCA: anti-neutrophil cytoplasmic antibody
BAFF: B-cell activating factor
BAFF-R: B-cell activating factor receptor
cAMP: 3',5'-cyclic adenosine monophosphate
FcRn: neonatal immunoglobulin Fc receptor
GO: Graves’ orbitopathy
ITP: immune thrombocytopenia
MG: myasthenia gravis
RTX: rituximab
SLE: systemic lupus erythematosus
TNF: tumor necrosis factor
TRAb: thyroid-stimulating hormone receptor autoantibody
TSH: thyrotropin (thyroid-stimulating hormone)
TSHR: thyroid-stimulating hormone receptor

## Additional Information

ORCiD numbers: 0000-0001-9661-0257 (X. Hu); 0000-0001-6748-4546 (Feifan Guo).

***Disclosure Summary:*** S.H.S.P. was chief investigator of the phase 1 trial of immunomodulatory TSHR peptide therapy. S.H.S.P. has received speaker fees from Quidel, Sanofi, Berlin Chemie. P.P. was a collaborator/subinvestigator for the peptide immunotherapy study.
